# Bisphenol a and the female reproductive tract: an overview of recent laboratory evidence and epidemiological studies

**DOI:** 10.1186/1477-7827-12-37

**Published:** 2014-05-09

**Authors:** Donatella Caserta, Noemi Di Segni, Maddalena Mallozzi, Valentina Giovanale, Alberto Mantovani, Roberto Marci, Massimo Moscarini

**Affiliations:** 1 Department of Gynecology-Obstetrics and Urological Sciences, “Sapienza”, University of Rome, S. Andrea Hospital, Rome, Italy; 2 Food and Veterinary Toxicology Section, Istituto Superiore di Sanità, Roma, Italy; 3 Department of Biomedical Sciences and Advanced Therapies, Section of Obstetrics and Gynaecology, University of Ferrara, Ferrara, Italy

**Keywords:** Bisphenol A, Endocrine disruptors, Fertility, Reproductive health, Genital tract

## Abstract

Bisphenol A (BPA) is a high production volume monomer used for making a wide variety of polycarbonate plastics and resins. A large body of evidence links BPA to endocrine disruption in laboratory animals, and a growing number of epidemiological studies support a link with health disorders in humans. The aim of this review is to summarize the recent experimental studies describing the effects and mechanisms of BPA on the female genital tract and to compare them to the current knowledge regarding the impact of BPA impact on female reproductive health. In particular, BPA has been correlated with alterations in hypothalamic-pituitary hormonal production, reduced oocyte quality due to perinatal and adulthood exposure, defective uterine receptivity and the pathogenesis of polycystic ovary syndrome. Researchers have reported conflicting results regarding the effect of BPA on premature puberty and endometriosis development. Experimental studies suggest that BPA’s mechanism of action is related to life stage and that its effect on the female reproductive system may involve agonism with estrogen nuclear receptors as well as other mechanisms (steroid biosynthesis inhibition). Notwithstanding uncertainties and knowledge gaps, the available evidence should be seen as a sufficient grounds to take precautionary actions against excess exposure to BPA.

## Background

There is growing concern regarding the impact of environmental pollutants on animal and human reproduction [1]. For instance, a substantial number of laboratory rodent studies suggest that the plasticizer Bisphenol A (BPA) may act as a reproductive toxicant, although many uncertainties remain as to the actual risk for humans [2]. BPA is one of the highest volume chemicals produced world-wide and is used in the manufacture of plastics and epoxy resins that are pervasive in our environment and in our daily lives [3]. BPA has estrogenic activity and binds to the α- and, to a lesser extent, β-estrogen receptors (ER) *in vitro* and *in vivo*[4]. Moreover, BPA can also inhibit the activity of endogenous estrogens and/or disrupt estrogen nuclear hormone receptor action [5]. There is additional clear evidence of a 17β-estradiol, BPA, and diethylstilbestrol (DES) membrane-binding site with a pharmacologic profile that differs from the classical ER. This membrane receptor may be implicated in triggering non-genomic actions of xenoestrogens and it has been demonstrated its existence both in pancreatic β cells and in pancreatic α cells [6].

BPA also affects other relevant endocrine activities, including the thyroid hormone pathway and binding to glucocorticoid and androgen receptors as well as more complex interferences with the central nervous system and immune system [3,5].

In experimental studies, BPA has been shown to affect many reproductive indices, such as ovary, uterus and vagina weights (indicating estrogenic action); egg shape; fertilization rate; number of live-born neonates per litter; distance between the genital pore and the anus in newborns (indicating the degree of masculinization or feminization of external genitalia); time of vaginal opening (indicating the start of female puberty); and onset of the estrous cycle (indicating sexual maturity) [2,7-9]. The evidence in the literature suggests that BPA exposure in humans may impair fertility [10].

In fact, we recently observed that women with BPA concentrations above the limit of detection were significantly more represented in the infertile population than in healthy controls [11]. In the last years, a few reviews have been published regarding the impact of BPA on human health [12-14]. These data are mainly derived from epidemiological studies, even though cross-sectional analyses cannot establish causality [15]. Furthermore, in human studies the possibility of an additive effect with other prevalent endocrine disrupting compounds should not be overlooked [13].

To the best of our knowledge, despite the vast literature of experimental animal and mechanistic studies, there are no reviews summarizing the toxicological studies regarding BPA’s effect on the female reproductive system. The aim of the present review is to synthetize and evaluate the experimental evidence regarding the impact of BPA on the female genital tract and to relate this evidence to the current knowledge of BPA’s effect on human reproduction.

In particular, we focused on the effect of BPA on the hypothalamic-pituitary-ovarian axis (HPOA), the ovary, the uterus and the development of diseases, such as polycystic ovarian syndrome and endometriosis.

## Methods

A systematic MEDLINE (PubMed) search was performed using the following keywords: “BPA”, “endocrine disruptors”, “fertility”, “reproductive health”, “genital tract”, “BPA and fertility”, “GnRH alterations and BPA”, “BPA and puberty onset”, “BPA and oocytes”, “BPA and primordial follicles”, “BPA and uterus”, “BPA and polycystic ovarian syndrome” and “BPA and endometriosis”. Relevant articles published in the last 10 years (from 2004 since November 2013) were selected. We considered experimental studies, epidemiological studies and previous reviews. Criteria for inclusion and exclusion were established before the bibliographic search. Studies published in languages other than English were considered if an English abstract was available. When evaluating experimental studies, we did not consider studies in which the effects of perinatal exposure were analyzed on the second generation. Moreover, studies in which BPA was administered with other endocrine disruptors were not considered as the aim of this study was to examine the potential direct association of BPA with female reproductive disorders; therefore, a discussion on the complex issue of the “cocktail” effects of endocrine disorders is outside the scope of this paper.

### BPA actions on the hypothalamic-pituitary-ovarian axis

During critical periods of prenatal and postnatal development, the hormonal milieu is crucial for the correct organization of neuroendocrine circuits that coordinate sex-specific physiology [16]. BPA exposure has been associated with alterations in brain sexual differentiation and modifications in the expression of gonadotropin-releasing hormone (GnRH), hypothalamic ER and luteinizing hormone (LH) [16-18].

Altered brain sexual differentiation has been demonstrated in perinatally treated rodents. In particular, BPA has been shown to alter the composition of the following two sexually dimorphic areas located in the hypothalamus: the sexually dimorphic nucleus (SDN) and the anteroventral periventricular nucleus (AVPV) [19]. BPA’s endocrine disrupting action could explain this effect; however, an epigenetic mechanism has been hypothesized as well [19]. Indeed, Kundakovic et al. [20] observed that in the hypothalamus of female offspring treated during gestation, the ER-α gene (Esr1) was hypomethylated for a downregulation of DNA methyltransferase 1 (DNMT1), an epigenetic regulator. However, unexpectedly, hypomethylation was associated with decreased Esr1 mRNA levels, suggesting that additional mechanism are involved in the transcriptional regulation of this gene [20].

The AVPV nucleus is fundamental for gonadotropin release and the LH surge [16-18]. The neural circuit that coordinates reproductive function is sexually differentiated; the female AVPV is nearly twice the size of the male AVPV, and neurons that express tyrosine hydroxylase (TH, rate-limiting enzyme for dopamine synthesis) are more abundant [17,19]. These sex differences are due to female development occurring in the absence of estrogen, whereas the perinatal male brain is exposed to high levels of estrogens synthesized locally through the aromatization of testicular testosterone [17]. Indeed, during critical periods of perinatal development, aromatase is present in specific brain regions [18].

Contrasting results have been reported regarding the effect of BPA on the number of TH-positive cells. Rubin et al. [18] observed a significant decrease in the number of TH-positive cells in the female population and no differences in males, while Patisaul [17] found an increased in the number of TH-positive cells in the male population. Such differences may be attributed to the species (rat or mouse in [17] and [18], respectively) or to the developmental exposure window (only neonatal or from organogenesis to neonatal in [17] and [18], respectively). AVPV-TH-containing neurons interact with GnRH fibers and modulate GnRH production [19]. GnRH secretion is pulsatile and acts on gonadotrope cells of the anterior pituitary to stimulate the synthesis and release of LH and follicle-stimulating hormone (FSH). The frequency of the GnRH pulses determines the predominant hormone produced. LH is produced with fast GnRH pulses, and FSH is favored with slow pulse frequencies [21]. Fernandez et al. [21] observed that female rats exposed to BPA during the neonatal phase had a higher GnRH pulse frequency and an increased number of peaks. Nevertheless, LH release in their study was lower basally and after GnRH stimulation, suggesting a qualitative, rather than quantitative, alteration in the gonadotrope hormone production. Monje et al. [16] even found that rats exposed in the neonatal period were incapable of producing an estradiol-induced LH surge. The decrease in basal and GnRH-induced LH release caused by BPA may be the consequence of the increased GnRH pulse frequency leading to a desensitization of the pituitary. Alternatively, a direct effect of BPA on LH secretion may occur [21]. Kisspeptins (KiSS) are critical hypothalamic regulators of GnRH secretion [22]. BPA exposure in rodents may alter the KiSS-regulation of the LH-surge system via a different mechanism that is dependent on the developmental window. Acute neonatal exposure seems to lead to downregulation [23], whereas prolonged perinatal (prenatal to neonatal) treatment seems to elicit an up-regulation of KiSS-positive AVPV cells in male rats [24]. Indeed, Xi et al. [25] observed that in mouse pups exposed to BPA both *in utero* (starting from gestation day 1) and postnatally (21–49), the expression levels of KiSS-1 mRNA were increased. Conversely, for pups exposed only postnatally (21–49) no noticeable changes in gene expression levels were detected, indicating a higher susceptibility during the prenatal window.

Overall, an influence of BPA-induced HPOA changes on the time of pubertal onset is plausible. Some studies have observed that early vaginal opening occurs in BPA-treated rodents (a sign of advanced puberty onset) [9,21]. This event may be explained by a premature hypothalamic-pituitary maturation induced by BPA treatment during the pre- and/or postnatal windows. However, the few human studies do not provide consistent results regarding a possible effect of BPA on pubertal development. Wolff et al. [26] investigated the association of exposure to phenols, phthalates, and phytoestrogens with pubertal stages in a multiethnic longitudinal study of 1,151 girls. They found that these endocrine-active agents had only limited association with pubertal development. In particular, the authors observed some weak association with phthalates and phytoestrogens but not with phenols. Conversely, in a case–control study by Qiao et al. [27], girls with premature puberty had significantly higher BPA serum levels. Therefore, the relationship between exposure to BPA and puberty onset in humans is still largely unknown. It should be noted that most animal studies were planned to gain mechanistic insights; thus, subcutaneous injection was often used, and in some cases, the administered doses were quite high. Upon real-life exposure by ingestion, dermal contact or inhalation, the majority of BPA is conjugated to BPA-glucuronide in the liver, which is a form devoid of interaction with nuclear receptors [28]. More experimental studies are needed to investigate BPA effects on HPOA and puberty onset using treatment routes and dose levels relevant to human environmental exposures. Table 1 shows BPA effects on the hypothalamic-pituitary-ovarian axis in animal experiments.

**Table 1:** BPA effects on the hypothalamic-pituitary-ovarian axis in animal experiments

**Study**	**Sample**	**Dose**	**Route of administration**	**Time of exposure**	**Results**
Monje et al. [16]	Female rats	0.05, 20 mg/kg day	Subcutaneous injections	PND 1-7	BPA20 group no production of estradiol-induced LH surge; ERα expression increased in AVPV and decreased in arcuate nucleus (ARC); PR expression decrease in AVPV
Patisaul et al. [17]	Rat offspring	250 μg every 12 h	Subcutaneous injections	PND 1-2	Increased number of TH-positive cells in the male population
Rubin et al. [18]	Mouse offspring	0, 25,250 ng/kg BW/day	Subcutaneous implantation (Alzet osmotic pumps)	GD 8- d 16 of lactation	BPA; 250 female offspring; significantly decreased number of TH-positive cells; alterations in sexually dysmorphic behavior
Kundakovic et al. [20]	Mouse offspring	2, 20, 200 μg/kg	Oral administration	GD O-19	Upregulation of Dnmt3a mRNA in the male prefrontal cortex correlated with hypermethylation of the Esr1 gene; downregulation of Dnmt1 in the female hypothalamus associated with hypomethylation of the Esr1 gene; decreased Esr1 expression in the male prefrontal cortex and in the female hypothalamus; changes in social, exploratory and anxiety-like behavior.
Fernandez et al. [21]	Female rats, pituitary cells in vitro	50, 500 μg/50 μl	Subcutaneous injections	PND 1-10	BPA500 significantly lower serum LH basally and after GnRH stimulation; alterations in GnRH pulsatility; advanced puberty onset
Navarro et al. [23]	Male and females rats	100, 500 μg/rat	Subcutaneous injections	PND 1-5	Decreased expression level of hypothalamic KiSS-1 mRNA at the prepubertal stage
Bai et al. [24]	Male rat offspring	2 μg/kg	Subcutaneous injections	GD 10- d 7 of lactation	Up-regulation of KISS-positive AVPV cells in prepubertal, pubertal, and adult male rats exposed perinatally to BPA
Xi et al., 2011 [25]	Mice offspring	12,25,50 mg/kg/day	By gavage	Dams: GD 1- PND 20; Pups: PND 21-49	Dose-dependent increases in the expression levels of KiSS-1, GnRH and FSH mRNA in BPA-exposed female and male pups; inhibition in the expressions of testicular steroidogenic enzymes and the synthesis of testosterone in the male pups; greater aromatase expression level and synthesis of estrogen in the female pups

### BPA actions on the ovary

As already noted for the actions on the HPOA, BPA has different effects on the ovary depending on the time of exposure.

Maternal exposure affects the earliest stages of oogenesis in the developing fetal ovary. The resulting meiotic defects increase the likelihood that embryos produced by the exposed females in adulthood will be chromosomally abnormal. In particular, several studies on *in utero* exposure of mice as well as rhesus monkeys revealed disturbances during the prophase events of meiosis and an increase in multiple oocyte follicles (MOFs) [29-31].

Disturbances during prophase events seem to mainly involve synapsis and recombination between homologous chromosomes. An interesting study by Susiarjo et al. [31] on pregnant mice exposed to BPA revealed synaptic abnormalities, such as “incomplete synapsis” (partial or complete synaptic failure of a single chromosome pair), end-to-end associations between non-homologous chromosomes and an increased risk of aneuploidy.

MOF occur due to improper cyst breakdown. In fact, in rodents, oocytes at birth are clustered in cysts and individual primordial follicles become detectable only when the cysts breakdown 3–5 days after birth. Usually, the cyst breakdown is initiated upon decreased estrogen levels after birth. BPA, acting as a synthetic estrogenic compound, may counteract this event [29,32,33]. Furthermore, gene ontology analysis suggests that BPA acts to downregulate genes involved in the mitotic cell cycle, raising the possibility that fetal BPA exposure may limit expansion of the primordial germ cell population [34].

Intrauterine exposure to BPA elicits analogous effects on early oogenesis and follicle formation in mice [29,31] as in rhesus monkeys [30]. This mammal is considered a valid model for human reproductive physiology [30]; hence, the findings of experimental studies raise concerns about the impact of BPA exposure on human reproductive programming.

Most studies conducted on rodents exposed to BPA during the early postnatal period report an initial follicle recruitment with a consequent decrease in the primordial follicle reserve as well as an increased incidence of MOFs [33,35,36]. These results were obtained even in a study conducted on lambs, a species in which the follicular development trajectory is similar to humans [32]. Follicle activation has been confirmed by Chao et al. [36], who found that upon BPA exposure, mouse ovaries had significantly decreased primordial follicles but increased primary, secondary and antral follicles. In contrast with other studies, Karavan et al. [33] observed an increased percentage of primordial follicles in neonatal mice injected with BPA during the first four postnatal days and examined on postnatal day 5. Table 2 shows effects of pre and postnatal BPA exposure on oocytes

**Table 2:** Effects on oocytes of prenatal and postnatal BPA exposure

**Study**	**Sample**	**Dose**	**Route of administration**	**Time of exposure**	**Results**
Zhang et al. [29]	Pregnant mice	0.02, 0.04, 0.08 mg/kg bw/day	Oral route	12.5-18.5 day post-coital	Inhibition of meiotic progression to prophase I in 0.08 BPA treated group; decreased mRNA expression of specific meiotic genes; inhibition of germ cell cyst breakdown
Hunt et al. [30]	Pregnant rhesus monkeys	400 μg/kg bw/day	tubing implants	GD 50–100, GD 100 to term	Disturbances in prophase events; increase in MOFs
Susiarjo et al.[31]	Pregnant mice, offspring	400 ng/day	pellets releasing BPA	GD 11.5-17.5	Aberrant meiotic prophase; increased aneuploidy in eggs and embryos from adult females
Rivera et al. [32]	Lambs	50 μg/kg/day	Subcutaneous injections	PND 1-14	Decreased ovarian weight; increased primordial-to-primary follicle transition; increased incidence of MOFs; increased number of small antral atretic follicles associated with higher p27 expression
Karavan et al. [33]	Mice	10, 100 μg/day	Subcutaneous injections	PND 1-4	Increased incidence of MOFs; increased total number oocytes; increased percentage of primordial follicles
Rodriguez et al. [35]	Rats	0.05, 20 mg/Kg/day	Subcutaneous injections	PND 1-7	In BPA 20 group stimulation of neonatal initial follicle recruitment; p27 and ERα increased expression; increased proliferation rate of granulosa cells
Chao et al. [36]	Mice	20, 40 μg/kg	Subcutaneous injections	PND 7–14, PND 5–20 (every 5 days)	Decreased methylation pattern of two maternal imprinted genes; upregulated mRNA expression of ERα; decreased primordial follicle number but increased primary, secondary and antral follicle number; abnormal spindle assembling in meiosis

In a recent study conducted by Lee et al. on adult female rats (8 weeks of age) exposed orally to 1 μg/kg body weight (BW) or 100 μg/kg BW for 90 days, caspase-3 activation was significantly increased by BPA exposure, causing an augmentation of follicular atresia and luteal regression [37]. BPA-induced apoptosis in granulosa cells has already been reported by Whetherill et al. [5], but the precise mechanism remains unclear. In addition, Lee et al. observed that 17β-estradiol synthesis was decreased via downregulation of aromatase. The steroidogenic acute regulatory protein (StAR) and aromatase cytochrome P450 (P450arom) appeared to be targeted by BPA [37]. The occurrence of negative effects on the ovary using lower doses of BPA (1 μg/kg BW), rather than the doses used in other experiments, should trigger further studies.

*In vitro* studies have demonstrated that BPA can interfere with ovarian hormone production.

A study conducted on antral follicles isolated from 32-day-old mice proved that BPA significantly decreased progesterone, dehydroepiandrosterone, androstenedione, estrone, testosterone, and estradiol production [38]. Because the BPA’s mechanism of action of is life stage-dependent, it cannot be excluded that BPA exposure close to or during sexual maturity mainly elicits an inhibiting action on steroid biosynthesis. This inhibitory effect has been observed even in isolated porcine granulosa cells [39] and in human luteal cells obtained from the corpora lutea of woman undergoing surgery for non-endocrine gynecological diseases [40]. In both studies, incubating cells with 0.1, 1, and 10 μM of BPA induced a significant decrease in progesterone production. Conversely, no inhibitory action was observed in a study by Dominuigez et al. [41] conducted on human granulosa-lutein cells collected from women undergoing in vitro fertilization (IVF) treatments. Instead, an increased metalloproteinase 9 (MMP-9) secretion was observed, suggesting a BPA effect on the stability of the ovarian extracellular matrix.

*In vitro* studies have also been conducted on human oocytes [42,43]. Genes involved in DNA double-strand generation, signaling and repair were up-regulated in fetal oocytes exposed to BPA [42]. Machtinger et al. [43] treated human oocytes obtained from patients undergoing IVF cycles with 0, 20, and 200 ng/ml BPA. In accordance with animal studies, they observed a negative effect on cell cycle progression, spindle architecture and chromosome organization.

The oocyte alterations observed in animal experiments and on human oocytes *in vitro* may explain the poor ovarian response to IVF treatments and the recurrent miscarriages observed in women with higher serum BPA concentration [12]. In particular**,** increased BPA levels are correlated with a decrease in peak estradiol levels and decreased oocyte retrieval number [10,44]. A similar mechanism of action may underlie the association between female infertility and higher BPA levels that has been consistently reported in the results of the Italian biomonitoring study *PREVIENI*[11,45]. Interestingly, in this study, the higher serum BPA levels in infertile women were also correlated with upregulation of some nuclear receptors (ERα, ERβ, Androgen receptor, and Pregnane-X receptor) in the serum mononuclear cells [11,45]. It is still unclear whether chemical compounds are linked with first-trimester spontaneous abortion [46]. Some studies reported a higher serum BPA concentration in women with recurrent miscarriages compared to healthy controls [46,47]. Zheng et al. [47] even observed that the risk of unexplained recurrent spontaneous abortion increased progressively with increasing serum BPA levels. Sugiura-Ogasawara et al. [46] suggested that the mechanism leading to an increased incidence of miscarriages from BPA exposure is an increase in embryonal chromosome abnormalities.

### BPA and its actions on the uterus

Mendoza-Rodriguez et al. [48] showed that when BPA was administered to rats in their drinking water (approximate dose 1.2 mg/kg BW/day) during pregnancy and lactation, there was a resultant significant increase in the thickness of the uterine epithelia and stroma in the adult female offspring. The majority of BPA-exposed offspring also had reduced epithelial apoptosis and a downregulation of α-estrogen receptor expression in epithelial cells during estrus. *In vi*tro studies show that BPA in other endometrial cells seems to induce different effects. Indeed, decreased proliferation was observed both in human endometrial fibroblasts isolated from hysterectomy specimens [49] and in human endometrial endothelial cells [50]. In particular, in the latter case, the authors found that mRNA levels of gene products involved in the cell cycle, cell division and cytoskeletal organization were affected [50].

Although BPA is not a genotoxic carcinogen, a possible endocrine-related tumorigenic effect might be elicited by BPA exposure during critical periods of differentiation. Atypical hyperplasia, stromal polyps of the uterus as well as sarcoma of the uterine cervix were observed in adult female offspring of mice injected with BPA during organogenesis. These lesions were completely absent in controls: however, the incidence was low and did not show a meaningful dose–response relationship; therefore, no conclusion could be drawn [51]. Nevertheless, the increased proliferative activity and reduction of programmed cell death in uterine epithelial cells upon intrauterine exposure, as observed in BPA-exposed rats by Mendoza-Rodriguez et al. [48], may support an increased predisposition to cancer and/or a tumor-promoting effect [48]. In contrast with these results, human epidemiological studies do not seem to support the relationship between BPA and endometrial disorders [12]. Indeed, Hiroi et al. [52] unexpectedly found that in patients with complex endometrial hyperplasia, serum BPA was significantly lower compared to both the control and simple endometrial hyperplasia groups. Even in postmenopausal endometrial cancer patients, serum BPA was significantly lower than either the control or simple endometrial hyperplasia groups.

There is a growing body of literature to suggest that BPA exposure may lead to defective uterine receptivity [53-55]. Varayoud et al. [53] observed a lower pregnancy rate and a reduction of implantation sites (live fetuses plus resorption sites) in female rats injected with BPA during the first postnatal week. Downregulation of homeobox A10 (Hoxa10) expression may explain, at least in part, the defective uterine receptivity. A different effect was observed by Bromer et al. [54] in mice from dams injected in utero during organogenesis (gestation days 9–16). In these cases Hoxa10 up-regulation and increased binding of ERα to the Hoxa10 Estrogen Responsive Element (ERE) were observed. As in other instances, it cannot be excluded that the discrepancy between effects observed in [53] and [54] is due to the different exposure windows, i.e., neonatal vs. prenatal, respectively. Alternatively, the drop in implantation rate could be due to a direct BPA effect on the development and transport of the pre-implantation embryo [55]. However, this possible explanation is less likely because the adverse effects of BPA mostly result from endocrine modulation rather than from target cell toxicity. An influence of BPA exposure on implantation success can also be surmised. Indeed, it has been observed that women with higher urinary BPA had higher implantation failure during IVF treatments [56]. Table 3 shows BPA effects on the uterus in animal experiments

**Table 3:** BPA effects on the uterus in animal experiments

**Study**	**Sample**	**Dose**	**Route of administration**	**Time of exposure**	**Results**
Mendoza-Rodriguez et al. [48]	Female rat offspring	1.2 mg/kg BW/day	BPA diluted in drinking water	GD 6- D 21 of lactation	BPA offspring: irregular estrous cycles; significant increase in the thickness of uterine epithelium and stroma; modifications in apoptotic patterns of uterine epithelium and ERα downregulation
Newbold et al. [49]	Mice	0.1 (BPA 0.1), 1 (BPA 1), 10 (BPA10), 100 (BPA 100), 1000 (BPA 1000) μg/kg/day	Subcutaneous injections	GD 9-16	Adenomatous hyperplasia in BPA1 and BPA100 not in controls; atypical hyperplasia in BPA 0.1, BPA 1, BPA 1000 not in controls; endometrial polyps in BPA 0.1, BPA 1, BPA 10; stromal sarcoma in one BPA 100 mouse
Varayoud et al. [53]	Female rats	0.05 (BPA 0.5), 20 (BPA 20) mg/kg day	Subcutaneous injections	PND 1,3,5,7	Pregnancies were not established in 10% of BPA 0.5 and in 23% of BPA 20, whereas in the controls the pregnancy rate was 100%; decreased number of implantation sites in BPA20; lower ERα and PR expression; lower expression of Hoxa10
Bromer et al. [54]	Female mouse offspring	5 mg/kg	Intraperitoneal injections	GD 9-16	Hoxa10 increased expression; decreased DNA methylation in the promoter and in intron regions of Hoxa10 with consequent increase in binding of ER-α to Hoxa10 ERE
Xiao et al. [55]	Pregnant mice	0, 0.025, 0.5, 10, 40, 100 mg/kg/day	Subcutaneous injections	GD 0.5-3.5	No implantation sites in BPA 100 treated group; smaller implantation sites, increased gestation period, reduced postnatal survival rate in 40 BPA treated group; alterations in PR expression.

### BPA and polycystic ovary syndrome (PCOS)

PCOS is the most common endocrinopathy among women of reproductive age. It is characterized by hyperandrogenism, insulin resistance and chronic anovulation. A role for BPA as an endocrine disruptor in the pathogenesis of PCOS has been recently proposed [57]. Several studies have reported higher BPA levels in premenopausal women with PCOS when compared to regularly ovulating women [57,58]. In addition, BPA treatment in rats, both during gestation or during the neonatal period, induced the development of a PCOS-like syndrome in adulthood [49,59].

A bidirectional mechanism has been hypothesized to underlie the relationship between PCOS and BPA, involving both BPA-increased insulin resistance and free androgen index as well as chronic, low-grade inflammation [57]. In fact, Kandaraki et al. [58] found a significant positive association between testosterone and androstenedione with BPA levels in women with PCOS. The increase in the androgen level may be explained by different mechanisms. The exposure of rat ovarian theca-interstitial cells to BPA *in vitro* results in elevated testosterone synthesis [58]. The increase in GnRH/LH pulse frequency observed in BPA experiments, as mentioned above, may be involved in this event [21]. Moreover, BPA alters androgen metabolism in the liver and acts as a potent ligand of sex hormone-binding globulin (SHBG); thus, it can displace androgens from SHBG resulting in increased levels of serum free androgens [58]. However, some studies have hypothesized that elevated BPA is a consequence and not a cause of PCOS because women with PCOS have higher circulating testosterone levels than do healthy women, and elevated androgen concentrations decrease BPA clearance [59].

### BPA and endometriosis

Endometriosis is a gynecological disorder characterized by endometrial glands and stroma that grow outside the uterine cavity. This ectopic endometrium responds to hormonal signaling; therefore, fetal exposure to estrogen is positively associated with endometriosis in adulthood [60,61].

Due to its ability to interact with ERs, BPA may be involved in the occurrence of estrogen-dependent pathologies. Strong epidemiological data link *in utero* exposure to the potent estrogen-mimicking drug DES and the occurrence of endometriosis later in adult life [60]; however, much less is known about exposure to BPA. In a pilot study, Cobellis et al. [62] investigated the possible relationship between serum levels of both BPA and the closely related, albeit much less investigated, bisphenol B (BPB) and the occurrence of endometriosis. These authors did not find BPA or BPB in any of the sera of healthy control women (n = 11). In contrast, the researchers found at least one of the bisphenols in 63.8% of sera of the 58 patients with endometriosis; 51.7% and 27.6% of these samples had detectable BPA or BPB levels, respectively, while 15.5% of patients had detectable levels of both bisphenols. In a study conducted on 465 women undergoing laparoscopy/laparotomy and 131 patients with endometriosis diagnosed by pelvic resonance imaging, Buck Louise et al. [63] found a positive association between urinary phthalates and endometriosis, while urinary BPA was not associated with endometriosis. In addition, Itoh et al. [64] analyzed the total urinary BPA concentration of 166 infertile patients and did not observe any association with endometriosis. It cannot be overlooked that different biomarkers of exposure may have different meanings. Therefore, the urinary BPA concentration (as used in [63] and [64]) provides information on the overall environmental exposure, while serum BPA [62] may be a more direct indicator of the internal exposure of target tissues, as outlined in the PREVIENI study on infertile Italian women [11,44].

A study on mice suggested a possible mechanism by which exposure to BPA may result in a predisposition to endometriosis. Signorile et al. [61] injected pregnant mice with BPA (100 and 1000 μg/kg/day) from day 1 of gestation to 7 days after delivery and found endometriosis-like structures in the adipose tissue surrounding the genital tract of a number of their sexually mature (3 month-old) offspring. Only one case was observed in the control group. Therefore, the authors suggested that endometriosis may be caused by alterations in the proper axial development of the Mullerian system during embryogenesis by changes in genetic-epigenetic programming. As previously mentioned, studies using parenteral routes of administration may have limited, or even no, relevance to human risk assessment. On the other hand, contrary to experimental studies, the human cross-sectional studies cannot give any insight on a possible effect on programming.

Considering the conflicting results, the hypothesis of a relationship between BPA and endometriosis needs to be further explored by robust and soundly designed human biomonitoring studies.

## Conclusions

An increasing number of studies indicate that exposure to BPA is associated with a variety of disorders of the female reproductive system. Toxicological risk assessment of the potential human health effects rely mainly on experimental studies that should be methodologically robust and provide information on exposure routes and levels relevant to humans but also on epidemiological studies, when available [15]. In this appraisal of the recent literature, we aimed to integrate and compare the results of both laboratory and human studies on BPA, which is among the most widespread endocrine disrupters. In relation to effects on the HPOA, animal experiments revealed alterations in brain sexual differentiation, higher GNRH pulsatility, alterations in the estradiol-induced LH surge and advanced puberty onset, as demonstrated by an early vaginal opening [16-18,21]. However, in women, the influence of BPA on the pathogenesis of premature puberty has not been confirmed. Some authors found a correlation between pubertal stages and the BPA serum level [27], while others observed conflicting results [26].

The higher risk of infertility and poor ovarian response to IVF treatments described in women with higher BPA concentrations suggest effects on oocyte quality and ovarian function in women [4,10,12,44,45]. In experimental studies, perinatal exposure has revealed disturbances in prophase events, and an increase in MOFs and initial follicle recruitment with increased numbers of primary, secondary and antral follicles [29-33,35,36]. *In vitro* studies on ovarian cells isolated from the ovary of both animals and women have also demonstrated an inhibiting action on steroid biosynthesis [38-40]. Thus, human and animal data show a degree of consistency.

BPA may reduce apoptosis and increase proliferation in the endometrium of perinatally exposed rats [48]; however, no tumorigenic effect of BPA on the uterus has been shown in adult women [52]. Additionally, rodent studies reported a defective uterine receptivity [53,55]; this event may be related to the higher risk of infertility or implantation failure observed during IVF treatments.

It has been suggested that BPA may be involved in the occurrence of some gynecological diseases. The association between BPA exposure and PCOS is supported by both human studies [57,58] and *in vivo* animal studies [49,59]. Indeed, BPA may induce an increase in androgen levels, while androgens decrease BPA clearance [57-59]. On the other hand, the involvement of BPA in the pathogenesis of endometriosis is doubtful; endometriosis-like structures were detected in mice perinatally injected with BPA [61]. Conversely, epidemiological studies did not yield consistent results [63,64]. Differences between laboratory evidence and epidemiological study results may be due to BPA dose, to the route of administration and to the time of exposure. Humans are exposed to BPA in a continuous manner and the main source of exposure is ingestion of contaminated food or water. Conversely, in most of the experimental studies reported in this review, BPA is administered only for a limited period through parenteral treatments. On the other hand, experimental studies allow a more detailed study of molecular events in target tissues, and, most important, allow for the investigation of long term effects of developmental exposures, which are important for BPA as well as for other endocrine disrupters. For obvious ethical reasons, controlled studies of the long-term exposure effects cannot be performed on pregnant women or children. The experimental studies provide some important information, namely, the BPA mode of action in some reproductive tissues may be highly dependent on the window of exposure [17,18,53,54]. The effects on HPOA [16-18,21], steroid biosynthesis in the ovary [37-40], or androgen-SBHG binding [57,58] clearly show that the potential harmful effects of BPA on female reproduction are more complex than “estrogenic” action. As for human biomonitoring studies, more insight would be gained by a consistent use of the biomarkers of exposure, e.g., urine vs serum [62-64]; the integration of the biomarkers of exposure with molecular markers of endocrine action [11,45,58]; and the use of bio-banks to investigate long-term effects.

Although the association between BPA exposure and some gynecological disorders is still doubtful, there is currently sufficient evidence to prompt precautionary actions against excess exposure to BPA.

## Abbreviations

AVPV: Anteroventral periventricular nucleus; BPA: Bisphenol A; BPB: Bbisphenol B; BW: Body weight; DES: Diethylstilbestrol; DNMT: DNA methyltransferase; ER: Estrogen receptor; ERE: Estrogen responsive element; Esr1: Estrogen receptor α gene; FSH: Follicle-stimulating hormone; GD: Gestational day; GnRH: Gonadotropin-releasing hormone; Hoxa10: Homeobox A10; HPOA: Hypothalamic-pituitary-ovarian axis; IVF: In vitro fertilization; KiSS: Kisspeptins; LH: Luteinizing hormone; MMP-9: Metalloproteinase 9; MOFs: Multiple oocyte follicles; PCOS: Polycystic ovarian syndrome; PND: Postnatal day; P450arom: Aromatase cytochrome P450; SDN: Sexually dimorphic nucleus; SHBG: Sex hormone-binding globulin; StAR: Ssteroidogenic acute regulatory protein; TH: Tyrosine hydroxylase.

## Competing interests

The authors declare that they have no competing interests.

## Authors’ contributions

DC**,** Massimo M and RM conceived the study, participated in the study design, compiled the contents and critically reviewed the paper. NDS was responsible for the literature (Medline) search, compilation of the information, drafting and finalizing the paper. MM, VG and AM provided substantial contributions, ranging from study ideas, design and a critical review of the final paper. All of the authors read and approved the final manuscript.

## Authors’ information

NDS, Maddalena M and VG are interns of the Department of Gynecologic-Obstetrical and Urological sciences, S. Andrea Hospital. AM is the director of the Food and Veterinary Toxicology Unit, Food Safety and Public Health Dept, Istituto Superiore Sanità, Roma. RM is a PhD, M.D and professor at “Ferrara University”. DC and Massimo M are PhD, M.D., and full professors at “Sapienza” University of Rome (Department of Gynecologic-Obstetrical and Urological sciences, S. Andrea Hospital). DC, AM and RM have previously collaborated on the “Previeni Project”, study in model areas on the environmental and health impact of some endocrine disrupters: living environment, reproductive outcomes and repercussions in childhood.
